# Climacteric complaints among very low-income women from a tropical region of Brazil

**DOI:** 10.1590/S1516-31802006000400008

**Published:** 2006-05-04

**Authors:** Sebastião Freitas de Medeiros, Márcia Marly Winck Yamamoto de Medeiros, Vivaldo Naves de Oliveira

**Keywords:** Climacteric, Social class, Hot flashes, Culture, Tropical climate, Climatério, Classe social, Fogachos, Cultura, Clima tropical

## Abstract

**CONTEXT AND OBJECTIVE:**

Climacteric symptoms may vary between different countries and cultures. Socioeconomic factors and climate may be implicated. The aim of this study was to identify climacteric symptomatology among very low-income Brazilian women, living in a hot and humid region.

**DESIGN AND SETTING:**

This cross-sectional population-based study was conducted in Cuiabá, at Júlio Müller University Hospital, a tertiary institution.

**METHODS:**

The study enrolled 354 climacteric women. The variables analyzed were social class, symptomatology and abnormal concurrent conditions. The study was approved by the hospital's research ethics committee.

**RESULTS:**

Sixty-five percent of the participants (232/354) were very poor and had had little schooling. The number of symptoms per woman was 8.0 ± 5.7. Hot flushes, nervousness, forgetfulness and fatigue were each found in nearly 60.0%. Tearfulness, depression, melancholy and insomnia were also frequent. Sexual problems were reported by 25%. The most relevant concurrent abnormal conditions reported were hypertension (33.9%), obesity (26.5%), arthritis/arthrosis (15.0%) and diabetes mellitus (9.6%). Hot flushes were associated with tearfulness, nervousness and forgetfulness.

**CONCLUSION:**

Brazilian climacteric women of low income and low schooling present multiple symptoms. Vasomotor and psychosexual symptoms were the most prevalent disorders. Hot flushes were associated with nervousness, forgetfulness and tearfulness.

## Resumo

**CONTEXTO E OBJETIVO:**

Sintomas climatérios podem mudar em diferentes culturas e países. Aspectos sócio-econômicos e clima podem estar envolvidos. Este estudo tem como proposta identificar os sintomas em mulheres climatéricas brasileiras de baixa renda de uma região quente e úmida do Brasil.

**TIPO DE ESTUDO E LOCAL:**

Estudo de coorte transversal, realizado no Hospital Universitário Julio Muller em Cuiabá.

**MÉTODOS:**

Este estudo incluiu todas as 354 mulheres climatéricas do Distrito Oeste de Cuiabá. Incluiuse na análise as variáveis classe social, sintomas e doenças coexistentes. Possíveis associações foram examinadas pelo teste χ^2^ e regressão logística.

**RESULTADOS:**

65% das pacientes eram muito pobres e de baixa escolaridade. O número médio de sintomas referidos por mulher foi de 8.0 ± 5.7. Fogachos, nervosismo, esquecimento e fadiga foram encontrados em quase 60% das mulheres. Choro imotivado, melancolia, depressão e insônia também foram freqüentes. Disfunções sexuais foram relatadas por cerca de 25% das mulheres. Fogachos foram associados com esquecimento, nervosismo e choro imotivado. As doenças coexistentes mais freqüentes foram hipertensão arterial (33,9%), obesidade (26,5%), artrite/artrose (15,0%) e diabetes *mellitus* (9,6%).

**CONCLUSÃO:**

Mulheres climatéricas brasileiras de baixa renda e baixa escolaridade são plurisintomáticas. Os sintomas vasomotores e psicosexuais foram os mais prevalentes. Fogachos foram associados a nervosismo, esquecimento e choro imotivado.

## INTRODUCTION

The main initial clinical manifestations of hypoestrogenism are hot flushes, sweating and palpitations. These are associated with fatigue, depression, anxiety, insomnia and forgetfulness. There is some evidence that this early climacteric symptomatology may vary between different countries and cultures. While in most Western societies their prevalence is high, in Oriental society and in other communities where the attitude towards the menopause is positive, the complaints appear to be fewer.^1^ For instance, Japanese women only present low levels of vasomotor symptoms associated with the menopause, and Indian women from Yucatan, in Mexico, may have no relevant symptoms at all.^2–4^ In Brazil, a tropical developing country, the prevalence of vasomotor symptoms in climacteric women has been reported to be as high as 74-84%.^5,6^

After a few years in a hypoestrogenic state, genitourinary atrophy appears. The most prevalent symptoms at this stage are vaginal dryness, dyspareunia, urinary incontinence and higher frequency of micturition. Worldwide, the reported rates of genital atrophic complaints are between 21% and 43% among postmenopausal women, and two out of three women aged of 75 present such complaints.^7,8^ Urinary incontinence has been reported to be between 7.4% and 73%, and it is more frequent among institutionalized women.^9,10^ In Brazil, there is a lack of information but, in a single study, dyspareunia was found in 31% and libido loss in 69% of a climacteric population.^11^

Because of the great variation in the prevalence of these symptoms, it has been suggested that they may be directly or indirectly influenced by preconceived attitudes towards the menopause, different exposure to stressful situations, lifestyle, race or socioeconomic status.

## OBJECTIVE

The majority of the current available data was obtained among non-Latin American women or among women of higher socioeconomic level. Here, we put forward the hypothesis that a hot and humid tropical environment may also be relevant. Therefore, the aim of this study was to investigate the extent of and characterize the climacteric symptomatology among urban, mixed race, very low-income women from a tropical region in a developing country.

## PATIENTS AND METHODS

This population-based, cross-sectional study was performed at the Júlio Müller Hospital, Universidade Federal de Mato Grosso, in Cuiabá, from June to December 2002. The prevalent symptoms and concurrent abnormal clinical conditions of 354 climacteric women, aged between 40 and 65 years, living in the western district of the city of Cuiabá, were analyzed. Formal consent was obtained from all patients for their participation in this study. The study followed the ethical criteria of the Brazilian Health Council and was approved by the Research Ethics Committee of the Júlio Müller University Hospital.

In determining the sample size, the study design took into consideration an 80% prevalence of hot flushes, type I error of 5% (α = 0.05) and 5% imprecision, i.e. a 95% confidence interval.^12^ Data were recorded on a previously adapted questionnaire used by the Department of Gynecology and Obstetrics of the Federal University of Mato Grosso, Brazil.^13^ The data analysis took into consideration the domains of the patients’ identification, anthropometry, medical history and symptomatology.

The sociodemographic variables of race, age, marital status, schooling and occupation were included. The social classes were determined by using the schooling level and purchasing power as the indicators, in accordance with the methodology of the Brazilian Association of Market Research Institutes.^14^ Briefly, this classification includes purchasing power, in which all household appliances owned are multiplied by a predetermined weight, the sanitary conditions in the home, and the schooling level. According to the number of points scored, the individual is placed in classes A, B, C, D, or E.

Associations between the variables considered were examined using the χ^2^ test. The strengths of the associations were estimated using the contingency coefficient (CC). Results were presented as odds ratios (OR) with 95% confidence intervals (95% CI). Possible interactions between hot flushes and other symptoms or abnormal clinical conditions were analyzed by logistic regression. Statistical significance in the comparisons was accepted when p < 0.05.

## RESULTS

The women's mean age was 49.7 ± 7 years. Most of them were married (65.8%), of mixed race (52%), and had had less than eight years of schooling (62.4%) or were illiterate (19.2%). About 84.3% were only occupied with domestic activities. Their mean body weight was 64.5 ± 13.6 kg and their mean body mass index (BMI) was 26.7 ± 5 kg/m^2^. Following the World Health Organization classification, 25% were obese and 4% were underweight.^15^

Nine percent (32/354) of these climacteric women belonged to social class B, 25% (90/354) to class C, 58% (206/354) to class D, and 7% (26/354) to class E. Sixty-five percent earned less than 250 United States dollars (US$) a month. Their characteristics are summarized in Table 1.

**Table 1.** Social and demographic characteristics of Brazilian climacteric women in Cuiabá

Characteristic	n	%
**Marital status**		
Single	37	10.5
Married	233	65.8
Widowed	48	13.6
Divorced	36	10.1
**Race**		
White	170	48.0
Black	83	23.5
Other	101	28.5
**Schooling**		
Illiterate	68	19.2
Primary school, unfinished	221	62.4
Primary school, complete	13	3.7
High school, unfinished	13	3.7
High school, complete	10	2.8
College/university	29	8.2
**Social class**		
A	00	0.0
B	32	9.0
C	90	25.4
D	206	58.2
E	26	7.4

The mean number of symptoms presented by each woman was 8.0 ± 5.7, ranging from none to a maximum of 19. Eighty-seven women (87/354; 24.6%) were asymptomatic, 41/354 (11.6%) reported one to five symptoms, 74/354 (20.9%) six to nine symptoms, 107/354 (30.2%) ten to fourteen symptoms, and 45/354 (12.7%) fifteen to nineteen symptoms. The proportions of all signs and symptoms reported are listed in Table 2. Nearly 60% of the women complained about hot flushes (206/354), nervousness (206/354) or forgetfulness (194/354), while more than 40% complained of tearfulness (153/354), depression (150/354), melancholy (148/354), insomnia (146/354), asthenia (174/354) and dizziness (153/354). Sexual problems were of concern for almost 25% of the women.

**Table 2.** Symptomatology reported by low-income Brazilian climacteric women in Cuiabá

Category	Symptoms/signs*	N	%†
Vasomotor	Hot flushes	206	58.2
	Palpitations	153	43.2
	Dizziness	153	43.2
	Sweating	120	33.9
Psychiatric	Nervousness	206	58.2
	Forgetfulness	194	54.8
	Tearfulness	153	43.2
	Depression	150	42.4
	Melancholy	148	41.8
	Insomnia	146	41.2
	Nostalgia	55	15.5
Sexual	Libido loss	111	31.1
	Dyspareunia/vaginal dryness	102	28.8
Urinary	Urinary incontinence	75	21.2
	Pollakiuria	69	19.5
Skeleton muscle	Myalgia	137	38.7
	Arthralgia	135	38.1
Others	Asthenia	174	49.2
	Formication	126	35.6
	Hair loss	123	34.7
	Headache	79	22.3
	Hair growth increase	37	10.5

* *More than one symptom per woman was reported;*

† *The percentage presented was estimated by considering all 354 women.*

At least one concurrent unfavorable clinical condition was reported by 266 of the women (75.1%). The most frequent associated diseases were arterial hypertension (120/354; 33.9%), obesity (33/354; 26.5%), arthritis/arthrosis (53/354; 15.0%), diabetes mellitus (34/354; 9.6%), heart disease (27/354; 7.6%), osteoporosis (25/354; 7.0%) and thromboembolic disease (7/354; 2.0%). Hot flushes were present in 90/120 (77.5%) of the hypertensive women and only in 135/254 (57.6%) of the normotensive women (p = 0.003). In the non-obese group, 175/259 (67.5%) complained of hot flushes and, in the obese group, 52/94 (55.3%) had this symptom (p = 0.034). No relationship was found between hot flushes and diabetes mellitus (p = 0.546) or type of menopause (p = 0.480).

There was an association between hot flushes and age, thus showing that this symptom was more prevalent among women aged over 49 (p < 0.001). In addition, hot flushes showed strong positive relationships (p < 0.05) with nervousness (OR: 38.2; 95% CI: 20.1-72.3), forgetfulness (OR: 15.7; 95% CI: 9.0-27.3), insomnia (OR: 15.1; 95% CI: 8-29.4) and libido loss (OR: 3.0; 95% CI: 1.7-5.1). However, logistic regression with adjusted odds ratios did not confirm the existence of any association between hot flushes and libido loss (p = 0.292; Table 3).

**Table 3.** Association between hot flushes and psychosexual complaints among low-income Brazilian climacteric women in Cuiabá

Psychosexual symptoms		Hot flushes		Bivariate analysis				Logistic regression		
		Yes	No	χ^2^	*CC	OR (95% CI)	^p^	aOR	95% CI	^p^
Nervousness	Yes	191 (83.8)	15 (16.2)	172.2	0.572	38.2 (20.1-72.3)	0.000	12.9	5.3-31.8	0.000
	No	37 (25.0)	111 (75.0)							
Insomnia	Yes	135 (92.5)	11 (7.5)	85.3	0.441	15.1 (7.8-29.4)	0.000	3.2	1.1-9.0	0.028
	No	93 (44.7)	115 (55.3)							
Forgetfulness	Yes	173 (89.2)	21 (10.8)	114.8	0.495	15.7 (9.0-27.3)	0.000	6.5	0.6-15.9	0.000
	No	55 (34.4)	105 (65.6)							
Libido loss	Yes	89 (80.2)	22 (19.8)	17.5	0.217	3.0 (1.7-5.1)	0.000	0.6	0.2-1.5	0.292
	No	139 (57.2)	104 (42.8)							

* *CC = contingency coefficient; OR = odds ratio; CI = confidence interval; aOR = adjusted odds ratio.*

## DISCUSSION

The present paper is the first to describe the climacteric symptoms among women living in the southern part of the Amazon region. This population living in a hot and humid region of Brazil was characterized by very low socioeconomic level and income of less than US$ 250/month. The number of symptoms reported by each woman in this Brazilian population was high. Nervousness, hot flushes, forgetfulness and asthenia were the most frequent complaints. The finding of at least eight symptoms per women is lower than what has been found among Chilean women,^16,17^ or in Pakistan, another developing tropical country, where the number of symptoms per women was even higher, and also related to the low socioeconomic level.^18^ The differences seen in these countries may involve culture, climate or even the different criteria used for determining social class.

Hot flushes were present in 58% of the women in the current study. Climacteric women from São Paulo city, with similar socioeconomic characteristics, presented a rate of hot flushes of between 74% and 84%.^6^ This high prevalence of vasomotor symptoms in Brazil does not differ from observations already made in several developed countries.^19^ Nevertheless, comparisons of the prevalence rates of hot flushes between countries have shown up remarkable differences that are currently attributed to dietary factors and cultural beliefs or social norms.^1^ Despite the high worldwide prevalence, low rates have been found in many Asian countries.^2^ Hot flushes are even absent among some Indian and Mexican groups. There is strong evidence that the diet consumed in Asian countries, which is rich in phytoestrogens, may decrease vasomotor symptoms. However, in some Asian countries with very similar diet, frequencies of hot flushes of up to 30-50% have been found.^20^ In Brazil, where the diet of low-income people is also rich in rice, beans and cassava (manioc) flour, a very high prevalence of hot flushes is seen nationwide. The bearing of this humid, high-temperature and muggy environment on the appearance of vasomotor symptoms has not been measured yet. Further studies evaluating the hypothesis that a tropical environment may modulate the appearance of hot flushes are needed.

Neuropsychiatric symptoms such as forgetfulness, nervousness and depression were seen in this study twice as frequently as reported from southern Brazil.^21^ While the population in the present study was poor and largely composed of non-white women, the women analyzed by Cassol in southern Brazil were essentially Caucasian European migrants and their descendants, of better socioeconomic level. Two other Brazilian studies, conducted in the south and on the southeast coast, demonstrated even more neuropsychiatric disorders. The differences are not clear yet, but the later studies were conducted in the most developed Brazilian states.^11,22^ The neuropsychiatric symptom rates found in these studies ranged from 40% to 70% did not, however, seem much higher than earlier reports from other cultures. In China, neuropsychiatric symptoms have been seen at a rate of up to 46%.^23^ In other cross-sectional studies, the rates of psychiatric manifestations among climacteric women have ranged from 25% to 40%.^24^ Although the menopause in itself does not appear to be the cause of such psychiatric modifications, changes related to the time around the menopause have been observed in up to 10% of the women included in prospective epidemiological studies.^25^

The sexual symptoms of loss of libido, dyspareunia and vaginal dryness were reported in one third of the climacteric women in the present study. A dyspareunia rate of as high as 31% had already been reported from southern Brazil.^22^ It has also been reported in 45% of Chilean climacteric women.^17^ Libido loss, dyspareunia, burning sensations and vaginal dryness are matters of concern for between 40% and 54% of climacteric women in the United States^26^ and Europe.^8^ Although the differences in prevalences are not clearly understood yet, they have also been attributed to different inclusion criteria, the women's ages and cultural factors.^26^ A distinction between urinary incontinence and urgency was not made in the present study, because of the epidemiological design. However the observed rate of 21% in the current report is in agreement with the majority of publications worldwide.

A few studies have examined factors that could predispose women to hot flushes, and it has been accepted that hot flushes can be modulated by psychosocial factors.^27,28^ Nervousness, forgetfulness, insomnia and libido loss were associated with vasomotor symptoms in the current study. In a logistic regression model, taking the combined effect of all these variables on hot flushes, only libido loss did not retain an independent association with vasomotor symptoms (adjusted OR: 0.6; 95% CI: 0.2-1.5).

## CONCLUSION

Most participants in this study had had little schooling and presented very low socioeconomic status, and they present multiple climacteric symptoms. These features of the sample studied may limit the findings from the current study to populations with the same characteristics. Women who are also sampled from the general population but who belong to higher social classes and have had greater schooling should also be studied in order to fully understanding the perception of symptoms relating to the menopause among Brazilian women. In addition, further studies focusing on the tropical environment and local dietary practices should be conducted in order to determine whether or not these variables might influence menopausal symptoms.

## References

[1] Medeiros SF, Oliveira VN, Yamamoto MMW (2003). Epidemiologia clínica do climatério. [Clinical epidemiology of climacteric]. Reprod Clim.

[2] Obermeyer CM (2000). Menopause across cultures: a review of the evidence. Menopause.

[3] Lock M, Kaufert P, Gilbert P (1988). Cultural construction of the menopausal syndrome: the Japanese case. Maturitas.

[4] Martin MC, Block JE, Sanchez SD, Arnaud CD, Beyene Y (1993). Menopause without symptoms: the endocrinology of meno-pause among rural Mayan Indians. Am J Obstet Gynecol.

[5] Baracat EC, Bortoletto CCR, Nunes MG (1995). Síndrome do climatério: aspectos epidemiológicos. GO.

[6] Halbe HW, Fonseca AM, Assis JS (1990). Aspectos epidemiologicos e clinicos em 1.319 pacientes climatericas. [Epidemiological and clinical findings in 1319 pos-menopausal woman]. Rev Ginecol Obstet.

[7] Berg G, Gottwall M, Hammar M, Lindgren R (1988). Climacteric symptoms among women aged 60-62 in Linköping, Sweden, in 1986. Maturitas.

[8] Stenberg A, Heimer G, Ulmesten U, Cnattingius S (1996). Prevalence of genitourinary and other climacteric symptoms in 61-year-old women. Maturitas.

[9] Barlow DH, Samsioe G, van Geelen JM (1997). A study of European womens’ experience of the problems of urogenital ageing and its management. Maturitas.

[10] Ouslander JG (1992). Geriatric urinary incontinence. Dis Mon.

[11] De Pelegrin GCL, Novaczyk AA, Winck CHL, Piovezan CM (1997). Avaliação dos sintomas clínicos das pacientes climatéricas atendidas no HUSM em relação ao uso de terapia de reposição hormonal e índice de massa corpórea. Reprod Clim.

[12] Katz DL, Katz DL (1997). Sample size, randomization, and probability theory. Epidemiology biostatistics and preventive medicine review.

[13] The North American Society Menopause Health Questionnaire.

[14] Mattar FN (1995). Análise crítica dos estudos de estratificação sócioeconômica de ABA-Abipeme. Revista de Admininstração.

[15] Deurenberg P, Yap M (1999). The assessment of obesity: methods for measuring body fat and global prevalence of obesity. Baillieres Best Pract Res Clin Endocrinol Metab.

[16] Blumel JE, Brandt A, Tacla X (1992). Perfil sintomatico de la mujer climaterica. Experiencia clinica. [Symptomatic profile of the climacteric female. Clinical experience. Rev Med Chil.

[17] Blumel MJE, Roncagliolo MME, Gramegna Sougarret GM, Tacla FX, Sepúlveda MH, Brandt Alvear A (1997). Prevalência de síntomas psiquicos y vasomotores en diferentes periodos del climaterio. [Prevalence of vasomotores and psychic symptoms in different periods of the climateric. Rev Chil Obstet Ginecol.

[18] Wasti S, Robinson SC, Akthar Y, Khan S, Badaruddin N (1993). Characteristics of menopause in three socioeconomic urban groups in Karachi, Pakistan. Maturitas.

[19] Whiteman MK, Staropoli CA, Benedict JC, Borgeest C, Flaws JA (2003). Risk factors for hot flashes in midlife women. J Women Health (Larchmt).

[20] Boulet MJ, Oddens BJ, Lehert P, Vemer HM, Visser A (1994). Climacteric and menopause in seven South-east Asian countries. Maturitas.

[21] Cassol G, Silva PB, Pinto CS (1997). Avaliação da depressão no climatério utilizando a escala de Zung. Reprod Clim.

[22] Almeida AB, Almeida SB, Rechden CL, Marques AL, Nervo CC (1997). Incidência dos sintomas das pacientes do ambulatório de climatério da ISCMPA. Reprod Clim.

[23] Liu P, He FF, Bai WP (2004). Menopausal depression: comparison of hormone replacement therapy and hormone replacement therapy plus fluoxetine. Chin Med J (Engl).

[24] Hunter M, Battersby R, Whitehead M (1986). Relationships between psychological symptoms, somatic complaints and menopausal status. Maturitas.

[25] Hunter MS (1992). Predictors of menopausal symptoms: psychosocial aspects. Baillieres Clin Endocrinol Metab.

[26] Bachmann GA (1995). Influence of menopause on sexuality. Int J Fertil Menopausal Stud.

[27] Freeman EW, Sammel MD, Lin H, Gracia CR, Kapoor S, Ferdousi T (2005). The role of anxiety and hormonal changes in menopausal hot flashes. Menopause.

[28] Binfa L, Castelo-Branco C, Blumel JE (2004). Influence of psycho-social factors on climacteric symptoms. Maturitas.

